# The influence of host genotype and salt stress on the seed endophytic community of salt-sensitive and salt-tolerant rice cultivars

**DOI:** 10.1186/s12870-018-1261-1

**Published:** 2018-03-27

**Authors:** Denver I. Walitang, Chang-Gi Kim, Kiyoon Kim, Yeongyeong Kang, Young Kee Kim, Tongmin Sa

**Affiliations:** 1 Department of Environmental and Biological Chemistry, College of Agriculture, Life and Environmental Sciences, Chungbuk National University, Cheongju, Chungbuk 28644 Republic of Korea; 2 Bio-Evaluation Center, KRIBB, Cheongju, 281-16 South Korea

**Keywords:** Community structure and diversity, Rice seed, Bacterial endophytes, Salt stress, Salinity tolerance, T-RFLP

## Abstract

**Background:**

Inherent characteristics and changes in the physiology of rice as it attains salt tolerance affect the colonizing bacterial endophytic communities of the rice seeds. These transmissible endophytes also serve as a source of the plant’s microbial community and concurrently respond to the host and environmental conditions. This study explores the influence of the rice host as well as the impact of soil salinity on the community structure and diversity of seed bacterial endophytes of rice with varying tolerance to salt stress. Endophytic bacterial diversity was studied through culture-dependent technique and Terminal-Restriction Fragment Length Polymorphism (T-RFLP) analysis.

**Results:**

Results revealed considerably diverse communities of bacterial endophytes in the interior of rice seeds. The overall endophytic bacterial communities of the indica rice seeds based on 16S rRNA analysis of clones and isolates are dominated by phylum Proteobacteria followed by Actinobacteria and Firmicutes. Community profiles show common ribotypes found in all cultivars of the indica subspecies representing potential core microbiota belonging to *Curtobacterium*, *Flavobacterium*, *Enterobacter*, *Xanthomonas*, *Herbaspirillum*, *Microbacterium* and *Stenotrophomonas*. Clustering analysis shows that the host genotype mainly influences the seed endophytic community of the different rice cultivars. Under salt stress conditions, endophytic communities of the salt-sensitive and salt-tolerant rice cultivars shift their dominance to bacterial groups belonging to *Flavobacterium*, *Pantoea*, *Enterobacter*, *Microbacterium*, *Kosakonia* and *Curtobacterium*.

**Conclusion:**

The endophytic communities of rice indica seeds are shaped by the hosts’ genotype, their physiological adaptation to salt stress and phylogenetic relatedness. Under salt stress conditions, a few groups of bacterial communities become prominent causing a shift in bacterial diversity and dominance.

**Electronic supplementary material:**

The online version of this article (10.1186/s12870-018-1261-1) contains supplementary material, which is available to authorized users.

## Background

Endophytic bacterial communities are very diverse within the plant structures. The density of populations as well as variations of endophytic bacteria generally decrease from the roots to the stems and leaves of the plants [1–4]. Diverse endophytic bacterial communities also thrive in the seeds of many plants [5–8]. Seed bacterial endophytes are especially interesting because of their intrinsic properties that allow them to colonize plant internal structures including the reproductive parts of the plants. Potentially the most intriguing characteristic of seed endophytes is their vertical transmission and conservation into the next generation plants [6, 8, 9].

Rice (*Oryza sativa* L.) harbors diverse genera of plant endophytes. Mainly diazotrophic communities have been investigated thoroughly across various rice cultivars [10, 11]. As bacterial endophytes colonize and establish in the rice endosphere, they would be affected by changes in plant physiology due to biotic and abiotic factors. Several studies in rice endophytes have already shown that changes during the maturation of the seeds also lead to shifts in competent bacteria composition [12]. Competent endophytes are microorganisms that successfully colonize and survive in the plant endosphere due to their inherent traits and adaptations [13]. To a greater degree, genotype determined the composition of the different endophytic bacterial communities across rice cultivars [14]. Seeds of plants also comprise the same major groups of bacteria found in the internal structures of roots, stems and leaves of plants. They belong to the major groups of α-, β- and γ-Proteobacteria, Firmicutes, Bacteroidetes and Actinobacteria [15] and are isolated from different seed sources [9], suggesting the widespread occurrence of seed endophytes. Hardoim et al. [8] also showed that rice seed endophytes are transmitted and seed-borne endophytes respond to the changes in the external environment and the growing conditions of their host plant.

Soil salinization due to the accumulation of salts, especially sodium chloride, affects agricultural lands and their vegetation even under irrigated landscapes [16]. With regards to plants, they have a time-dependent response to salinity starting with water stress effects and finally resulting to altered flowering time, reduced seed production and even growth termination [17]. Generally, rice is moderately sensitive to salinity but some cultivars are salt tolerant to a certain degree allowing them to sustain growth under salt stress conditions [18]. Sensitive cultivars accumulate ions more quickly than tolerant cultivars [17] with tolerance mechanism involving exclusion or reduction of Na uptake and increased absorption of K [18]. Though there are physiological differences between salt-sensitive and salt-tolerant rice cultivars, both are still affected especially during high salinity and prolonged exposure. In the quest to increase physiological tolerance to salinity, heterosis as a result of a hybrid between a high yielding salt-sensitive and a salt-tolerant but low yield rice plants is an effective alternative [19]. Establishing patterns in community structure and diversity of bacterial endophyte in the hybrids may also help us understand if there are potential correlations between host tolerance and its native endophytic microbiota.

Seed-associated microbial communities are in general understudied. Even fewer studies have attempted to correlate endophytic bacteria of seeds with their hosts’ genotypes, physiological adaptation to salt stress and host phylogenetic relatedness. There are also no studies on the impact of salt stress on transmissible seed endophytic community up to this date, especially on rice cultivars with a differing degree of tolerance to salinity. In this study, a comprehensive analysis of the seed bacterial community will be done through T-RFLP analysis and 16S rRNA sequencing of bacterial isolates and clone libraries to gain insights into the seed endophyte diversity within six rice cultivars. The effect of salt stress was also investigated on the seeds of physiologically different cultivars grown under different soil salinity levels. The objective is to compare the structure and diversity of bacterial endophytes in the rice seeds of several indica subspecies as influenced by the inherent differences in their host characteristics and then by external factor particularly the effect of salt stress.

## Methods

### Seed samples for studying seed bacterial community of the six rice cultivars

To compare the community structure and diversity of the different rice cultivars and to see potential influence of the host characteristics to the seed endophyte community, six rice cultivars were selected. All seeds of the six different cultivars used in this study were cultivated and taken from Rural Development Administration (RDA), South Korea (Additional file 1: Table S1). There were six genotypes included in the study representing different cultivars. There were five salt-tolerant cultivars included namely IR669646-3R-178-1-1 (FL478), CSR 28 (IC27), IR55179-3B-11–3 (IC31), IR58443-6B-10-3 (IC32), IRRI154 (IC37), and a salt-sensitive cultivar (IR29). The salt-tolerant cultivars: IC27, IC31, IC32 and IC37 are experimental hybrid lines being studied for their salinity tolerance. IR29 is a sodium accumulating line that is salt-sensitive during early and mature plant growth development. FL478 is a hybrid from the salt-sensitive line IR29 and a salt-tolerant sodium excluding Pokkali [20]. IC27 and IC31 have one common parent, IR4630–22–2-5-1-3 which is also distantly related to Pokkali. One of the parents of IC32 could also be traced to Pokkali. IC37 have parental lines different from the other cultivars in the study. All cultivars in this study belong to *Oryza sativa ssp. indica*. Seed germination started in May 2014 and seedling transplantation was done in June. All the seed samples were harvested from August to early September 2014. Composite samples of each rice cultivars were made from the harvested plants growing in the fields. All seeds used for DNA and bacterial isolation were fresh. Seeds were also stored at 4 °C without any seed treatment for further use.

### Surface sterilization of rice seeds

Bacterial communities of the rice seed endosphere from the six cultivars were also assessed by culture-dependent approaches. Surface sterilization of rice seeds was done according to Hardoim et al. [8]. Under sterile conditions, decontaminated forceps were used to remove the hulls of rice seeds (1 g). Subsequent surface-sterilization was done at 30 °C for 25 min in an orbital shaker (200 rpm) with a 50 ml solution containing 0.12% sodium hypochlorite (NaClO) and salts (0.1% sodium carbonate, 3% sodium chloride, and 0.15% sodium hydroxide) [21]. Removal of the surface adhered NaClO was achieved by washing with 50 ml 2% sodium thiosulfate [22] repeated twice at 30 °C for 10 min under orbital shaking (200 rpm). The seeds were rinsed 5–8 times with sterile distilled water before the seeds were subjected to rehydration for at least 1 h at room temperature in 100 ml autoclaved demineralized water. The efficiency of sterilization was confirmed by plating 100 μL of the final rinse onto R2A agar plates and incubating them for 7 days at 28 °C.

### Culturable microbial population

Surface sterilized seeds were ground with an autoclaved mortar and pestle. Culturable populations of seed endophytic bacteria were determined by counting the colony forming units (CFU) on R2A (DB – Difco) plates using spread plate technique after serial dilution of the homogenized surface sterilized seed samples (1.0 g). Ten-fold serial dilutions were made and 100 μl aliquots were spread onto an R2A agar in three replicates for each dilution. Plates were incubated at 28 °C. For bacteria population, counting was done every 24 h for 6 days. Unique bacteria from each plate were chosen based on colony color and morphology. Identification of the bacterial isolates was done through 16S rRNA gene sequence analysis.

### Seed bacterial endophytes of salt-sensitive and salt-tolerant rice cultivars under salt stress conditions

From the six rice cultivars, three were chosen to represent rice host with varying levels of physiological tolerance to salt stress: salt-sensitive, IR29; moderately salt-tolerant, IC32; highly salt-tolerant, IC37. In addition to physiological tolerance to salt stress, the three cultivars were also chosen since they have the most diverse endophytic community and that they are not directly linked through parental lineage. Surface sterilization of the rice seeds was done as mentioned above. The seeds were germinated aseptically on 4.405 g L^− 1^ Murashige and Skoog medium (Duchefa) amended with 4 g L^− 1^ Phytagel (Sigma) for 3 days. Germinating seeds were transferred in seedling trays or seed beds. About 21 days to one month after seeding, 5 seedlings were transferred in plastic bins with 4.5 kg paddy soil (chemical characteristics: pH 6.1; electric conductivity (EC) 0.65 dS m^− 1^; organic matter 1.28 g kg^− 1^ dry soil; cation exchange capacity (CEC) 1.53 cmol (p+); total N content 0.03%; available P_2_O_5_ 31.34 mg kg^− 1^ dry soil; K 0.3 cmol kg^− 1^ dry soil; Ca^2+^ 0.21 cmol kg^− 1^ dry soil; Mg 0.44 cmol kg^− 1^ dry soil and Na 0.11 cmol kg^− 1^ dry soil). The soil was collected from the paddy fields of Chungbuk National University, South Korea. The non-autoclaved paddy soil was also amended with N, P_2_O_5_ and K_2_O according to RDA’s fertilizer application rates for paddy fields (N 110 kg ha^− 1^; P_2_O_5_ 45 kg ha^− 1^ and K_2_O 57 kg ha^− 1^). The plants were maintained in green house condition at Chungbuk National University, Korea. After two weeks of transplantation, seedlings were thinned to 3 per pot. The water level was maintained about 1 cm above the soil. After 3 months of vegetative growth, salt stress was imposed on the rice plants at two levels, 4 dS m^− 1^ (~ 40 mM NaCl) and 8 dS m^− 1^ (~ 80 mM NaCl). Salt concentration was calculated based on the amount of soil used. For example, a total of 10.5174 g of NaCl was added to 4.5 kg of soil to achieve 40 mM NaCl concentration. To avoid osmotic shock, the salt solution was added gradually by putting 10 mM NaCl to each pot daily, and the desired salt concentration was achieved after 8 days for 80 mM NaCl. One liter of water was used to completely saturate the soil in each pot with water. Water saturation point of the soil was maintained throughout the day during salt stress imposition. Five replicate pots per cultivar per salinity level were prepared for the experiment. Composite samples of mature seeds from replicate pots were harvested, dried for three days under direct sunlight and stored at 4 °C for DNA extraction. Seed germination started in April, salinity was imposed in July, and the seeds were harvested in late August to early September 2016.

### 16S rRNA gene sequence analysis

Pure cultures of endophytic bacteria were subjected to 16S rRNA sequence analysis. Isolates were grown on nutrient agar plates. Genomic DNA was extracted and PCR was used to amplify the 16S rRNA genes using the primers 27F: 5′-AGA GTT TGA TCC TGG CTC AG-3′ as the forward primer and 1492R: 5′-GTT TAC CTT GTT ACG ACT T-3′ as the reverse primer [23] followed by identification of the 16S rRNA nucleotide sequences using PCR-direct sequencing, via the fluorescent dye terminator method (ABI Prism™ Bigdye™ Terminator cycle sequencing ready reaction kit v.3.1). The products were purified using Millipore-Montage dye removal kit and ran in an ABI3730XL capillary DNA sequencer with a 50 cm capillary. The obtained 16S rRNA sequences were aligned and the affiliations deduced using BLAST analysis in the EzTaxon server (https://www.ezbiocloud.net/) [24]. Phylogenetic analyses were performed using MEGA version 6 [25] after multiple alignments of the data by CLUSTAL W [26]. DNA substitutions were done according to the Jukes and Cantor model [27] and clustering was performed using the neighbor-joining method [28]. The statistical confidence of the nodes was estimated by bootstrapping using 1000 replications [29]. The nucleotide sequences of 16S rRNA genes were deposited in the GenBank® database under accession numbers KY393309-KY393357.

### Total DNA extraction

Total genomic DNA extraction of seeds was done according to Johnston-Monje and Raizada [6] with minor modifications. One gram of surface-sterilized seeds for each genotype was ground in an autoclaved mortar and pestle. One mL of 50 mM Na_2_HPO_4_ buffer per gram of seed dry weight was added. Total genomic DNA was extracted from 0.1 g of extract using DNeasy Plant Mini Kits (Qiagen, USA) following manufacturer’s protocol. DNA concentration was also quantified by using Nanodrop2000 (Thermo Scientific).

### PCR amplification for T-RFLP

PCR conditions for amplification of bacterial DNA were done according to Johnston-Monje and Raizada [6]. A PCR mastermix was made with the following components: 2.0 μl Standard Taq Buffer, 0.8 μl of 25 mM each of dNTP mix, 0.5 μl of 10 μM 27 F-Degen primer with sequence AGRRTTYGATYMTGGYTYAG [30] (where R = A + G, Y = C + T, M = A + C), 0.5 μl of 10 μM 1492R primer with sequence GGTTACCTTGTTACGACTT [30], 0.25 μl of Standard Taq, 20 ng of total DNA, and the final volume was made up to 20 μl with double distilled water. Amplification was performed for 25 cycles in a PTC200 DNA Thermal Cycler (MJ Scientific, USA) using the following program: 96 °C for 3 min, 25× (94 °C for 30 s, 48 °C for 30 s, 72 °C for 1 min 30 s), 72 °C for 7 min.

To increase the total DNA amplification, the volume of the reaction mix was made to 50 μl for the second nested PCR. The reaction mixture consisted of 5.0 μl Standard Taq Buffer, 4.0 μl of 25 mM each of dNTP mix, 2.0 μl of 799f primer, 2.0 μl of 1492R primer, 0.3 μl of Standard Taq, 2.0 μl of 10^− 1^ PCR product from the first PCR reaction, and double distilled water up to 50.0 μl total. For the nested PCR, primer 799f with sequence AACMGGATTAGATACCCKG [31] (where M = A + C, K = G + T), was labeled with 6FAM, and 1492R primer with sequence GGTTACCTTGTTACGACTT [30] was used. The forward primer 799f was chosen as it is strongly biased against amplifying chloroplast 16S rRNA [31]; the much larger mitochondrial 18S fragments were later removed in silico after amplification and restriction. Amplification was performed for 25 cycles in a PTC200 DNA Thermal Cycler (MJ Scientific, USA) using the following program: 95 °C for 3 min, 25× (94 °C for 20 s, 53 °C for 40 s, 72 °C for 40 s), 72 °C for 7 min.

### Seed bacterial 16S rRNA clone library generation and sequencing

Fifty μL of each PCR product was run on an electrophoresis gel, and the 730 bp (bacterial 16S) and the ~ 850 bp (mitochondrial DNA) were gel extracted and ligated to PCR cloning vector (T-Blunt, Solgent) according to the manufacturer’s instructions. Thirty clones from each transformation were screened by colony PCR using plasmid primers M13 Forward and M13 Reverse, combined with amplified ribosomal DNA restriction analysis (ARDRA) [32] with DdeI and HhaI. Clones with distinct restriction patterns were subjected to colony PCR sequencing using M13 Forward and M13 Reverse and sequenced, identified and analyzed as with bacterial isolates. Clone sequences were submitted to Genbank under accession numbers KY862075 – KY862113. To predict fragment sizes from clones and cultures, sequences were submitted to the in silico T-RFLP analysis program TRiFLe [33].

### Restriction enzyme digestion

PCR purification products were digested separately using three restriction enzymes: DdeI, HaeIII and HhaI. For the restriction enzymes, 0.8 μl of 4 U each, 2 μl 10× buffer (buffer C for HaeIII and HhaI), 2 μl of 10× BSA and MilliQ water, adjusted according to the volume of the PCR purification product (1.0 μg/μl) with a total volume of 20 μl. Digestions with HaeIII, HhaI and DdeI enzymes were carried out at 37 °C, for 16 h. All enzymes and reagents were from Promega, USA. The separation and detection of digestion products were carried out by electrophoresis using 2% QA-agarose TM gel. Five μl of the enzyme digestion products and 6× dye were loaded on the agarose gel.

### Sizing

To determine the precise length of the Terminal Restriction Fragments (T-RFs), 1.5 μl digests were mixed with 9 μl Hi-Di™ formamide (ABI) and 0.6 μl of size standard (500ROX, Bioventures). The samples were denatured at 95 °C for 3 min, then placed on ice for 5 min. Sizes of restriction fragments were determined on an automated ABI 3130 DNA sequencer (Applied Biosystems, USA). Fluorescently labeled 5′ T-RFs were detected and analyzed by using Genemapper, ver. 3.7 (Applied Biosystems), with size mapper (500 ROX). DNA extraction, amplification, restriction and analysis were repeated twice for all seed samples of the six rice cultivars and the results were merged together. T-RFLP analysis for the effect of salt stress was only done once.

### Analysis

T-RF peaks identified from individual T-RFLP profiles were compiled, arranged and adjusted for statistical analysis. To compensate for differences in the PCR product quantity and T-RFLP profile intensity among samples, the relative abundance was calculated based on the peak area of each sample divided by the sum of all peak areas from the corresponding sample [34]. Richness (S) was determined by the presence or absence of RF bands in the electrogram. Shannon diversity index (H′) was determined using the formula *H′* = −∑(*p*_*i*_)(ln *p*_*i*_), while Shannon evenness (J’) was calculated as J’ = H′/ln(S), and Simpson index as (1/D) = 1/∑pi^2.^ In these equations, *p*_*i*_ is for the relative abundance of T-RFs, ln is for the natural log, S is for the number of species and D is for Simpson’s dominance index, which is inversely proportional to diversity. Comparison of diversity indices between the treatments was done by one-way ANOVA and Tukey’s test using SAS (Ver 9.4). Data were tested for normality and homoscedasticity with Shapiro-Wilk test and Levene’s test, respectively, using STATISTICA (version 8.0, Statsoft, USA) statistical package. Some data were power-transformed (from Y^2^ to Y^4^) to meet the assumption of homogeneity of variances required for ANOVA. Differences between the mean were analyzed with Kruskal-Wallis test and Dunn’s test using STATISTICA statistical package when the data did not meet the assumption for ANOVA despite the data transformation.

T-RFLP data set were analysed by nonmetric multidimensional scaling (NMDS) using primer V.6 software package. Briefly, each T-RFLP data set was imported into the Primer V.6 and a similarity matrix was calculated using Bray Curtis coefficient. The MDS procedure was then used to ordinate the similarity data following 100 random starts. Goodness-to-fit or stress was calculated using Kruskal’s stress formula: **Stress** = √Σ_h*,i*_ *(d*_*hi*_*-ď*_*hi*_*)*^*2*^*/Σ*_*h,i*_*d*^*2*^_*hi*_), where, *d*_*hi*_ is the ordinated distance between samples *h* and *i*, and *ď* is the distance predicted from the regression. The ANOSIM (analysis of similarities; PRIMER 6) was used to assess significant differences in the profile composition. NMDS ordinations and associated ANOSIM tests were run for each digestive enzyme. Visualization of the relative abundance using heatmaps was done using matrix2png interface (http://www.chibi.ubc.ca/matrix2png/bin/matrix2png.cgi).

## Results

### Rice seed endophytic community across different cultivars

Six rice cultivars grown under normal soil conditions at the experimental fields of RDA (South Korea) were selected and harvested in accordance with their characteristics. The composition and diversity of their seed endophytic communities were investigated in relation to plant genotype, host phylogenetic relatedness and salinity tolerance using culture-dependent technique as well as T-RFLP culture-independent technique.

**Fig. 1:**
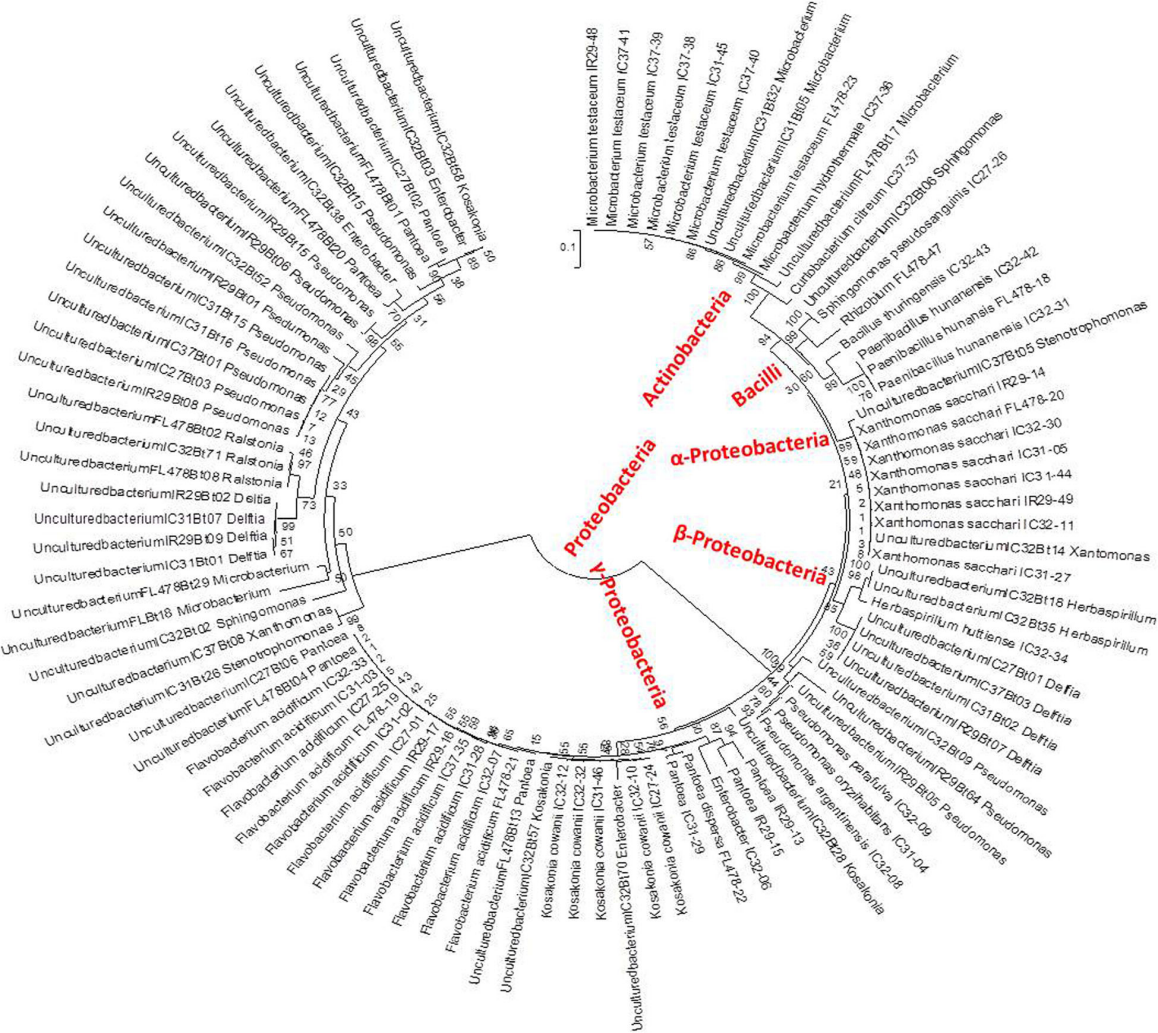
Phylogenetic tree of bacterial 16 s rRNA sequences from *Oryza sativa ssp. indica* seed endophyte clones (Uncultured bacterium_clone_putative genus) and isolates (Closest match_bacterial strain)

**Fig. 2:**
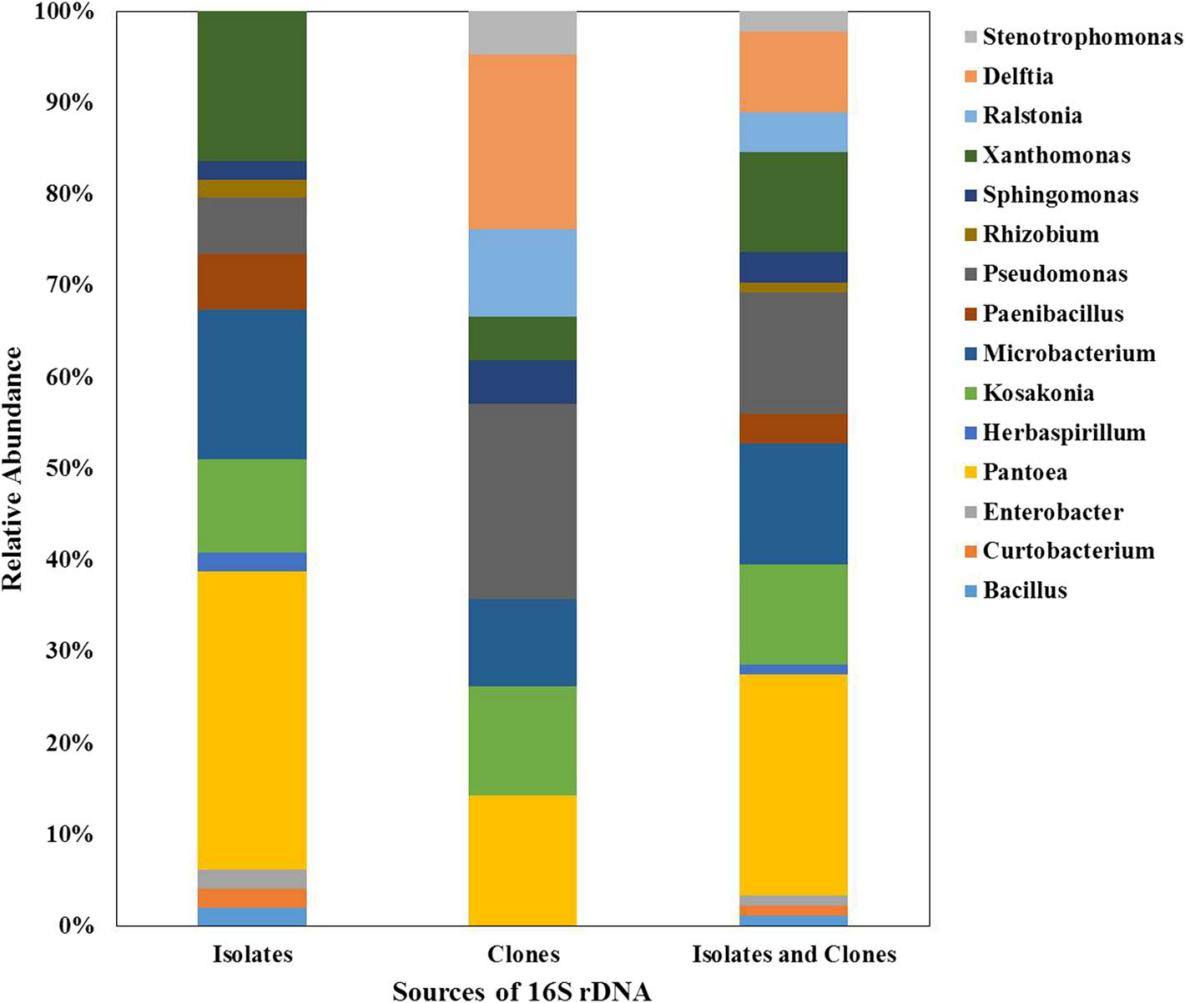
Relative abundance of the different endophytic bacterial genera based on sequenced bacteria isolated from seeds (isolates) and amplified 16 s rRNA gene clone library (clones)

The population of the culturable bacterial endophytic community of rice seeds was assessed in the salt-tolerant and salt-sensitive cultivars (Additional file 2: Table S2). There were 18 distinct bacterial species encompassing members of 12 genera within the classes Alpha, Beta and Gamma Proteobacteria, Actinobacteria and Bacilli. Combining both isolates and clone libraries showed that the endophytic bacterial communities of the indica rice seeds were dominated by the Phylum Proteobacteria (82%) followed by Actinobacteria (14%) and by Firmicutes (4%) (Fig. 1). In the genus level, *Pantoea* dominated (22%) followed by *Pseudomonas* (16%), *Microbacterium* (13%), *Kosakonia* (10%) and *Xanthomonas* (10%) (Fig. 2).

T-RFLP analysis of the rice seed endophytes showed considerable complexity in their bacterial diversity comparatively shown in the community profiles produced using three restriction enzymes DdeI, HaeIII and HhaI. Diversity indices varied among cultivars but showed generally similar trends observed in all the T-RFLP profiles (Table 1). Richness ranged from 5 to 15, 8–11 and 11–15 T-RFs for DdeI, HaeIII and HhaI digestion, respectively, where the salt-sensitive strain IR29, generally had a higher number of ribotypes compared to most of the other salt-tolerant cultivars. The diversity among the moderately and strongly tolerant rice was less predictable and most probably attributed to inherent differences in the cultivars rather than to physiological adaptation to salt stress although significant differences occurred across the cultivars.

**Table 1:** Diversity indices of bacterial endophytes inhabiting the seeds of different rice cultivars after digestion with restriction enzymes based on T-RFLP analysis

Diversity parameter	Rice cultivar	T-RFLP Restriction Enzyme Profile		
		DdeI	HaeIII	HhaI
Richness	IR29 (SS)	12.33 ± 2.6^a^	10.00 ± 0.58^a^	14.00 ± 1.00^a^
	FL478 (MT)	9.00 ± 0.01^a^	10.33 ± 0.33^a^	11.33 ± 0.88^a^
	IC27 (MT)	13.00 ± 2.0^a^	9.00 ± 0.58^a^	11.33 ± 0.33^a^
	IC31 (MT)	5.33 ± 0.67^a^	8.67 ± 0.33^a^	11.33 ± 0.33^a^
	IC32 (MT)	7.00 ± 0.00^a^	8.33 ± 0.33^a^	11.67 ± 0.33^a^
	IC37 (ST)	13.33 ± 0.33^a^	8.67 ± 0.33^a^	11.00 ± 0.00^a^
Evenness	IR29 (SS)	0.70 ± 0.02^ab^	0.88 ± 0.01^ab^	0.91 ± 0.02^a^
	FL478 (MT)	0.73 ± 0.01^a^	0.78 ± 0.20^cd^	0.88 ± 0.01^ab^
	IC27 (MT)	0.82 ± 0.04^a^	0.92 ± 0.00^a^	0.90 ± 0.03^a^
	IC31 (MT)	0.58 ± 0.05^b^	0.74 ± 0.02^d^	0.87 ± 0.01^ab^
	IC32 (MT)	0.80 ± 0.01^a^	0.88 ± 0.01^ab^	0.92 ± 0.00^a^
	IC37 (ST)	0.80 ± .0.01^a^	0.83 ± 0.01^d^	0.81 ± 0.01^b^
Shannon index	IR29 (SS)	1.71 ± 0.16^ab^	2.02 ± 0.06^a^	2.40 ± 0.05^a^
	FL478 (MT)	1.61 ± 0.02^ab^	1.82 ± 0.02^ab^	2.13 ± 0.07^bc^
	IC27 (MT)	2.10 ± 0.21^a^	2.02 ± 0.06^a^	2.18 ± 0.04^b^
	IC31 (MT)	0.95 ± 0.04^b^	1.59 ± 0.05^c^	2.11 ± 0.01^bc^
	IC32 (MT)	1.56 ± 0.02^ab^	1.87 ± 0.04^ab^	2.27 ± 0.03^ab^
	IC37 (ST)	2.07 ± 0.04^a^	1.80 ± 0.02^bc^	1.93 ± 0.04^c^
Simpson’s index	IR29 (SS)	0.73 ± 0.04^ab^	0.84 ± 0.01^a^	0.89 ± 0.01^a^
	FL478 (MT)	0.74 ± 0.01^ab^	0.77 ± 0.02^bc^	0.86 ± 0.01^ab^
	IC27 (MT)	0.80 ± 0.05^ab^	0.85 ± 0.01^a^	0.87 ± 0.01^ab^
	IC31 (MT)	0.48 ± 0.05^b^	0.72 ± 0.02^c^	0.85 ± 0.00^b^
	IC32 (MT)	0.72 ± 0.01^ab^	0.82 ± 0.01^ab^	0.88 ± 0.00^ab^
	IC37 (ST)	0.83 ± 0.01^a^	0.79 ± 0.00^ab^	0.80 ± 0.01^c^

Values given are the means of three replicates ±standard error. Values of the same letter are not statistically significant at *p* < 0.05. *SS* salt-sensitive cultivar, *MT* moderately tolerant cultivar, *ST* strongly tolerant cultivar

### The occurrence of common ribotypes and genotype-specific ribotypes across cultivars, phylogenetically related hosts and hosts with differences in physiological adaptation to salt stress

**Fig. 3:**
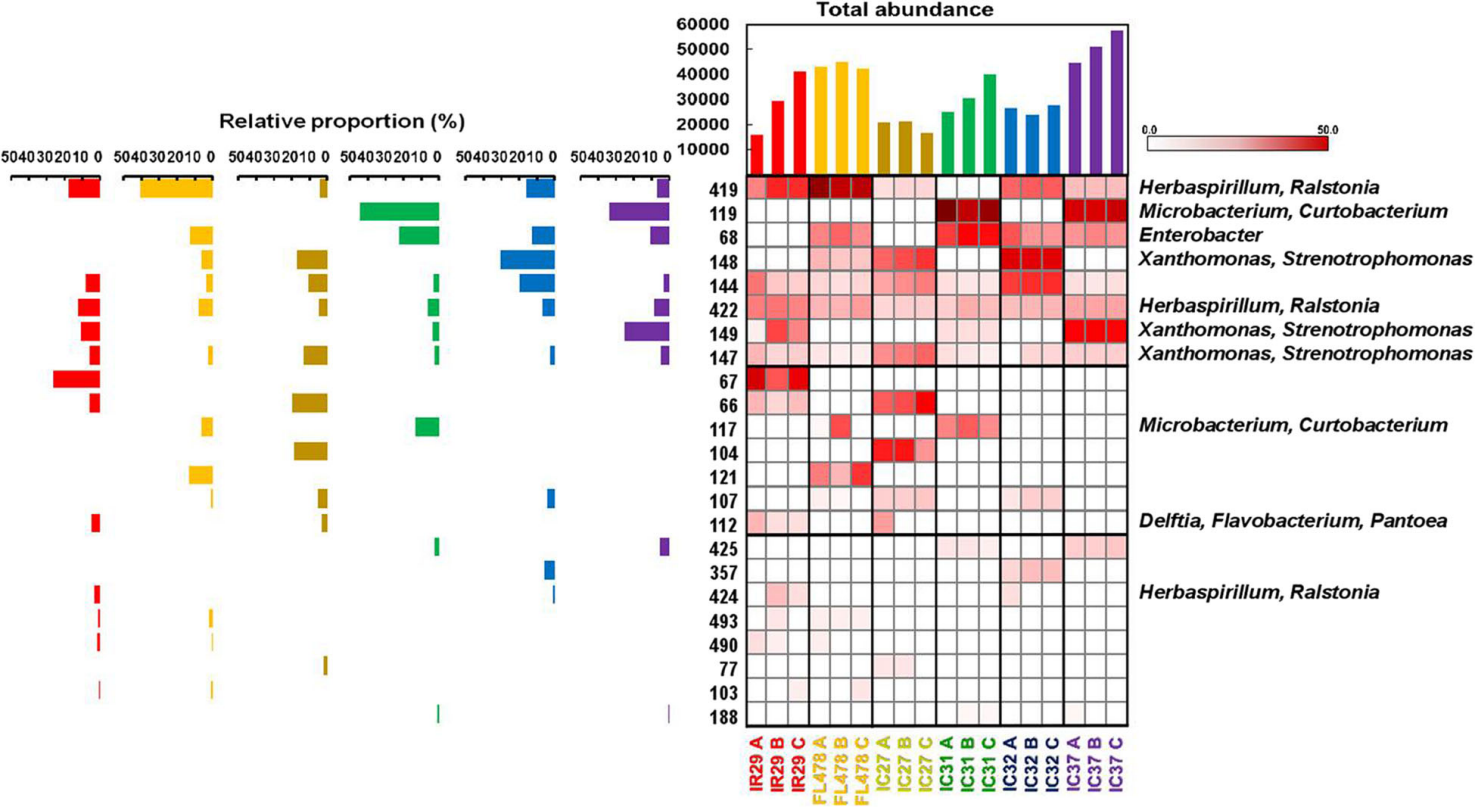
Heat map, relative abundance and total abundance of ribotypes (T-RF’s) present in the seeds of different rice cultivars after digestion with HaeIII. IR29 is a salt-sensitive cultivar, FL478, IC27, IC31 and IC32 are moderately salt-tolerant cultivars and IC37 is a highly salt-tolerant rice cultivar. T-RFs are on the left side of the heat map and the identities of the T-RFs were based on in-silico prediction of 16S rRNA sequences of isolates and clones from the study. The heat map is arranged according to decreasing overall abundance

T-RFLP analysis of the rice seeds using DdeI, HaeIII and HhaI restriction enzymes showed patterns in occurrences of common T-RFs or ribotypes as well as genotype-specific T-RFs across cultivars, phylogenetically related hosts and hosts with differences in their physiological tolerance to salt stress (Fig. 3, Additional file 3: Figure S1, Additional file 4: Figure S2). The common ribotypes seemed to be independent to the different factors and appear across the indica subspecies being considered in this study. On the other hand, occurrences of unique ribotypes were mainly attributed to the specific genotypes of rice to where they were observed.

T-RFLP profiles showed common T-RFs observed across cultivars in all three restriction enzymes. DdeI had three (64, 506, 355), HaeIII had three (144, 422, 147) and HhaI showed three (353, 322, 189) common ribotypes present in all rice cultivars. The identity of the T-RFs traced using both culturable and clone libraries of the amplified 16S rRNA genes showed that the common T-RFs are members of the genera *Curtobacterium* (506), *Xanthomonas* (347 and 346) and *Delftia* (64) using DdeI restriction enzymes. In the HaeIII digests, T-RF 422 belongs to *Herbaspirillum* while T-RF 147 is attributed to *Xanthomonas* and *Stenotrophomonas*. The common T-RF, 353, observed in HhaI profile is attributed to *Xanthomonas* and *Microbacterium*. On the other hand, *Flavobacterium*/*Pantoea* signals were separately detected in most cultivars except IC31 (Fig. 3, Additional file 3: Figure S1, Additional file 4: Figure S2) in the different T-RFLP profiles; although *Flavobacterium* strains were isolated from IC31 (Fig. 1).

The presence of commonly shared T-RFs was observed in higher occurrence among salt-tolerant rice cultivars compared to biologically related cultivars. Candidate ribotypes only found among salt-tolerant cultivars included T-RFs 240 and 347 (DdeI) attributed to *Pantoea*, *Flavobacterium* and *Xanthomonas*; T-RF 68 (HaeIII) identified as *Enterobacter*; and T-RFs 75 and 177 (HhaI) belonging to *Sphingomonas* and *Pseudomonas,* respectively.

There was only one ribotype uniquely found between the parent-daughter cultivar (IR29 and FL478) in Hha I (T-RF 82) and none in the half-siblings (IC27 and IC31). There were also a few ribotypes found only in the progeny cultivars of Pokkali (FL478, IC27 and IC32) including T-RFs 104 and 148 and T-RF’s 461, though they were not observed in the other Pokkali progeny, IC31.

The occurrence of unique T-RFs, those that are only found on a specific cultivar, is a characteristic of that cultivar, and their occurrence may be due to more complex factors (Table 2). In all the T-RFLP profiles based on the different restriction enzymes used, IR29 consistently have the most number of unique T-RFs with 40%, 33.3% and 33.3% occurrence in DdeI, HaeIII and HhaI, respectively. The unique T-RFs among the moderately salt-tolerant cultivars ranged from 0 to 42.9%, while that of the highly tolerant cultivar ranged from 0 to 21.4%. Though occurrence of unique T-RFs may be affected by host phylogenetic relatedness and physiological adaptation to salt stress and other factors, each genotype seems to acquire their own genotype-specific T-RFs. They probably occurred based on inherent differences of the cultivars regardless of their host phylogenetic relatedness, physiological similarities or other attributing factors.

**Table 2:** Summary of unique (genotype-specific) ribotypes found in the seeds of different rice cultivars based on the T-RFLP profiles after digestion with DdeI, HaeIII and HhaI

Genotype	DdeI			HaeIII			HhaI		
	Unique T-RF	Total T-RF	% Unique	Unique T-RF	Total T-RF	% Unique	Unique T-RF	Total T-RF	% Unique
IR29	6	15	40	3	9	33.3	5	15	33.3
FL478	2	9	22.2	2	10	20	1	12	8.3
IC27	6	14	42.9	2	9	22.2	2	12	16.7
IC31	1	6	16.7	0	8	0	3	12	25
IC32	1	7	14.3	1	9	11.1	1	12	8.3
IC37	3	14	21.4	0	8	0	1	11	9.1

### The community structure of seed bacterial endophytes with respect to dominant T-RFs

The community structure of bacterial endophytes in the rice seeds in terms of distribution and equal representation of the different T-RFs based on abundance is significantly different in the various cultivars (Table 1). Some cultivars such as IC32 and IC27 consistently showed an even distribution of ribotypes and that the abundance of its individual T-RFs was not extreme. IC31 consistently showed a significantly lower evenness indicating that there are T-RFs which are more dominant in terms of abundance. IC37 also showed a significantly lower evenness, seen in HaeIII and HhaI T-RFLP profiles, compared to most of the cultivars in the study. Fig. 3, S1 Figure and S2 Figure were arranged mainly based on the abundance of the fragments or ribotypes. The most abundant T-RFs could be the dominant bacterial communities in each cultivar and potentially affected the processes during the developmental stages of the host plant. Interestingly, there were also a few dominant T-RFs that are found in all the different cultivars such as fragment 64 (*Delftia*) in DdeI, 144 (unidentified) and 422 (*Herbaspirillum*) in HaeIII and 353 (*Xanthomonas*, *Microbacterium*) and 322 (unidentified) in HhaI. There are also a few T-RFs that are unique and dominant on a specific cultivar.

### Rice seed endophytic communities influenced by genotypes, physiological adaptation to salt stress and host phylogenetic relatedness

**Fig. 4:**
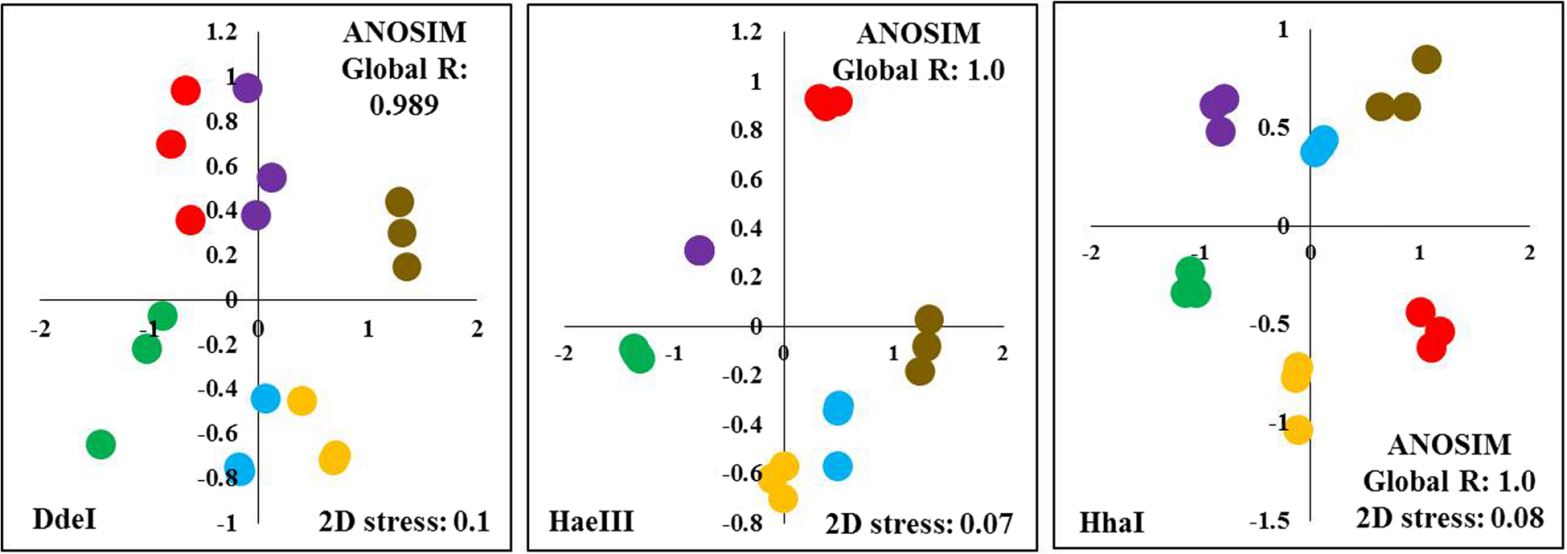
NMDS ordination of the endophytic bacterial community T-RFLP after digestion with restriction enzymes Dde I, Hae III, and HhaI.  IR29 (Red),  FL478 (Orange),  IC27 (Gold),  IC31 (Green),  IC32 (Blue),  IC37 (Purple). Global R values were calculated through Analysis of Similarity (ANOSIM). *P*-values of ANOSIM for DdeI, HaeIII and HhaI are significant at P(%) = 0.1

To compare overall similarities and differences in endophytic bacterial populations between the different rice cultivars based on T-RFLP profiles and abundances, NMDS analysis and cluster analysis were done (Fig. 4 and Additional file 5: Figure S3). Distances between points in the NMDS ordination reflect relative similarities of bacterial populations among samples. Cultivars that are more similar to one another are ordinated closer. Digestion with restriction enzymes DdeI, HaeIII and HhaI showed a distinct separation of endophytic bacterial communities in all cultivars. A value of 1 or close to 1 for global R of all the rice samples as well as R values between cultivars suggested dissimilarities between groups and indicate the dependence of the bacterial structure to the individual genotype or cultivar. Kruskal’s stress values were lower than or equal to 0.1 in all T-RFLP profiles suggesting a fair representation of the relationships between points in the NMDS matrix. Distinct separation of bacterial communities based on cultivars was also clearly established by doing Analysis of Similarity test evaluating dissimilarities of T-RFLP profiles of the different cultivars.

Combining results of NMDS and cluster analysis showed the patterns of grouping among rice cultivars (Fig. 4 and Additional file 5: Figure S3). Each cultivar formed distinct clusters indicating that genotype is the major effector shaping the endophytic bacterial communities of the indica seeds. Effects of host phylogenetic relatedness could also be seen in some cultivars though results are not as consistent when strictly comparing with the family tree of the cultivars included in the study. The parent-daughter effect of IR29 and FL478 was only observed in Hha I where they are ordinated more closely together. The cousins FL478 and IC32 were consistently ordinated closely together compared to the other cultivars showing the potential contribution of their common parental lineage (Pokkali) and physiological similarity to salt stress tolerance. On the other hand, the half-siblings IC27 and IC31were consistently ordinated far from one another as opposed to the expectation that they would have higher similarity due to their common parent, IR460–22–2-5-1-3. The effects of physiological adaptation to salt stress seemed to show a greater contribution to the clustering and ordination of the rice cultivars, after genotype. T-RFLP profile based on Dde I showed that the moderately tolerant cultivars IC32, FL478 and IC31 were more closely ordinated with IC27 as an outlier. Ordination of HaeIII and HhaI community profiles showed a separation of the cultivars based on salinity tolerance where cultivars were generally grouped according to their salinity tolerance as salt-sensitive, moderately tolerant and highly tolerant cultivars. *P*-values of ANOSIM for DdeI, HaeIII and HhaI are significant at P(%) = 0.1 (Additional file 6).

Though each rice cultivar has a distinct set of endophytic seed bacterial community, overall clustering and similarities of their microbiota showed that the indica subspecies of rice included in this study altogether have a high degree of commonality. Group similarity of DdeI and HaeIII T-RFLP profiles show higher than 30% while HhaI profile has greater than 40% overall similarity (Additional file 5: Figure S3).

### Rice seed endophytic communities influenced by salinity of the soil

Rice seeds coming from cultivars with varying degrees of tolerance to salinity had bacterial endophytes that responded differently when their host plants experienced different levels of soil salinity. The diversity of seed endophytes of the salt-sensitive cultivar IR29 decreased under 4 dS m^− 1^ and 8 dS m^− 1^ when compared to the non-stress condition (Table 3). On the other hand, the bacterial endophytes of the moderately tolerant IC32 and the highly tolerant IC37 showed a different response. Moderate soil salinity stress at 4 dS m^− 1^ seems to have induced a more even distribution of bacterial abundance, albeit no significant changes in terms of species richness in IC32. This could have led to a higher diversity index under moderate salinity in IC32 as partly observed in HaeIII and HhaI community profiles. Interestingly, the highly salt-tolerant cultivar IC37, generally showed significantly higher richness, evenness and Shannon Index under moderate salinity. High salinity at 8 dS m^− 1^ has reduced evenness as well as diversity indices of seed endophytes in the salt-tolerant rice cultivars, IC32 and IC37 (Table 4 and Table 5).

**Table 3:** Diversity indices of seed bacterial endophytes of the salt-sensitive IR29 grown in varying soil salinity level as assessed through T-RFLP analysis

Diversity Parameter	Salinity Level	T-RFLP Restriction Enzyme Profile		
		DdeI	HaeIII	HhaI
Richness	0 dSm^−1^	8.67 ± 0.33^B^	8.67 ± 1.20^A^	8.33 ± 0.67^A^
	4 dSm^−1^	3.67 ± 0.67^C^	5.33 ± 1.86^A^	5.67 ± 0.33^C^
	8 dSm^−1^	10.0 ± 1.15^A^	6.33 ± 0.33^A^	6.67 ± 0.33^B^
Evenness	0 dSm^− 1^	0.82 ± 0.00^A^	0.81 ± 0.04^A^	0.80 ± 0.02^B^
	4 dSm^− 1^	0.69 ± 0.07^A^	0.77 ± 0.04^A^	0.87 ± 0.03^A^
	8 dSm^−1^	0.70 ± 0.01^A^	0.75 ± 0.03^A^	0.54 ± 0.01^C^
Shannon Index	0 dSm^−1^	1.77 ± 0.04^A^	1.73 ± 0.04^A^	1.69 ± 0.08^A^
	4 dSm^−1^	0.85 ± 0.02^C^	1.18 ± 0.21^A^	1.50 ± 0.04^A^
	8 dSm^−1^	1.59 ± 0.05^B^	1.37 ± 0.03^A^	1.03 ± 0.04^B^
Simpson’s Index	0 dSm^−1^	0.77 ± 0.01^A^	0.78 ± 0.03^A^	0.77 ± 0.02^A^
	4 dSm^−1^	0.48 ± 0.03^C^	0.62 ± 0.05^C^	0.73 ± 0.02^B^
	8 dSm^−1^	0.73 ± 0.01^B^	0.66 ± 0.01^B^	0.49 ± 0.02^C^

Values given are the means of three replicates ±standard error. Values of the same letter are not statistically significant at *p* < 0.05

**Table 4:** Diversity indices of seed bacterial endophytes of the moderately salt-tolerant IC32 grown in varying soil salinity level as assessed through T-RFLP analysis

Diversity Parameter	Salinity Level	T-RFLP Restriction Enzyme Profile		
		DdeI	HaeIII	HhaI
Richness	0 dSm^− 1^	11.33 ± 0.88^A^	5.33 ± 0.33^A^	6.67 ± 0.33^A^
	4 dSm^−1^	5.67 ± 0.33^C^	6.00 ± 0.00^A^	5.67 ± 0.33^A^
	8 dSm^−1^	9.00 ± 0.58^B^	5.33 ± 0.33^A^	7.00 ± 1.00^A^
Evenness	0 dSm^−1^	0.71 ± 0.01^B^	0.72 ± 0.01^B^	0.68 ± 0.01^B^
	4 dSm^−1^	0.75 ± 0.02^AB^	0.87 ± 0.01^A^	0.74 ± 0.02^A^
	8 dSm^−1^	0.83 ± 0.00^A^	0.70 ± 0.03^C^	0.61 ± 0.05^C^
Shannon Index	0 dSm^−1^	1.71 ± 0.04^B^	1.20 ± 0.06^A^	1.28 ± 0.01^A^
	4 dSm^−1^	1.30 ± 0.07^C^	1.55 ± 0.02^A^	1.27 ± 0.04^B^
	8 dSm^−1^	1.81 ± 0.05^A^	1.16 ± 0.01^A^	1.15 ± 0.02^C^
Simpson’s Index	0 dSm^−1^	0.76 ± 0.01^AB^	0.63 ± 0.03^A^	0.63 ± 0.01^B^
	4 dSm^−1^	0.66 ± 0.03^B^	0.77 ± 0.01^A^	0.66 ± 0.01^A^
	8 dSm^−1^	0.81 ± 0.00^A^	0.60 ± 0.01^A^	0.57 ± 0.01^C^

Values given are the means of three replicates ±standard error. Values of the same letter are not statistically significant at *p* < 0.05

**Table 5:** Diversity indices of seed bacterial endophytes of the highly salt-tolerant IC37 grown in varying soil salinity level as assessed through T-RFLP analysis

Diversity Parameter	Salinity Level	T-RFLP Restriction Enzyme Profile		
		DdeI	HaeIII	HhaI
Richness	0 dSm^− 1^	6.67 ± 1.45^B^	6.00 ± 0.00^C^	6.67 ± 0.33^A^
	4 dSm^−1^	11.0 ± 1.53^A^	8.67 ± 0.88^A^	6.67 ± 0.67^A^
	8 dSm^−1^	8.67 ± 1.20^AB^	6.33 ± 0.88^B^	6.67 ± 0.88^A^
Evenness	0 dSm^−1^	0.90 ± 0.04^A^	0.80 ± 0.01^AB^	0.75 ± 0.02^B^
	4 dSm^−1^	0.74 ± 0.01^B^	0.89 ± 0.02^A^	0.84 ± 0.02^A^
	8 dSm^−1^	0.54 ± 0.02^C^	0.45 ± 0.00^B^	0.33 ± 0.01^C^
Shannon Index	0 dSm^−1^	1.64 ± 0.14^B^	1.43 ± 0.02^C^	1.42 ± 0.02^A^
	4 dSm^−1^	1.87 ± 0.14^A^	1.91 ± 0.05^A^	1.58 ± 0.06^A^
	8 dSm^−1^	1.16 ± 0.07^C^	0.82 ± 0.06^B^	0.62 ± 0.56^B^
Simpson’s Index	0 dSm^−1^	0.79 ± 0.02^A^	0.72 ± 0.01^A^	0.69 ± 0.01^A^
	4 dSm^−1^	0.78 ± 0.02^A^	0.84 ± 0.00^A^	0.77 ± 0.01^A^
	8 dSm^−1^	0.50 ± 0.03^B^	0.37 ± 0.02^B^	0.26 ± 0.02^A^

Values given are the means of three replicates ±standard error. Values of the same letter are not statistically significant at *p* < 0.05

**Fig. 5:**
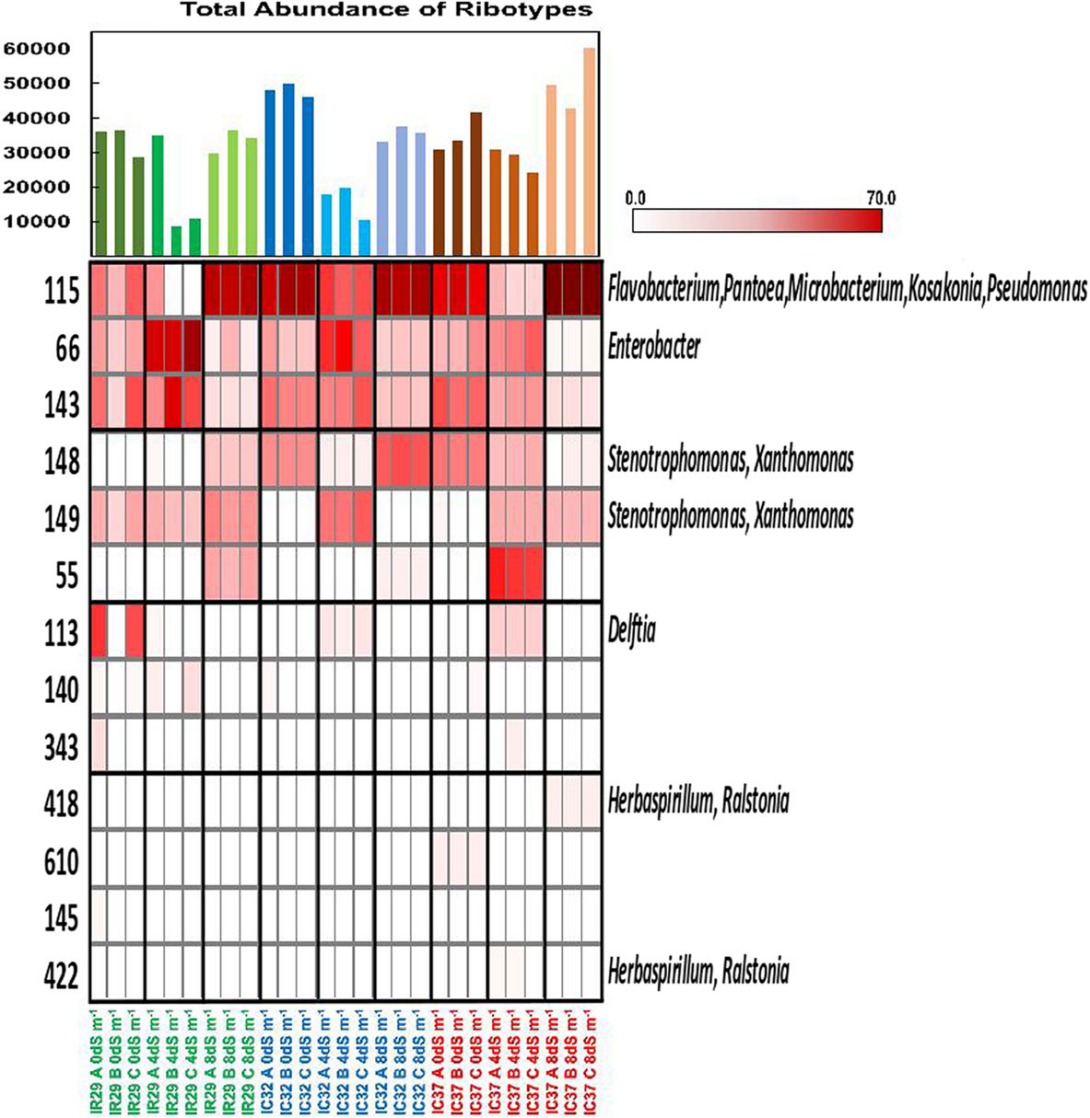
Heat map, relative abundance and total abundance of ribotypes (T-RF’s) after HaeIII digestion present in the seeds of three rice cultivars based on their tolerance to salt: IR29 – salt sensitive, IC32 – moderately tolerant, IC37 – highly tolerant, grown under normal condition (0 dS m^− 1^), moderate salinity stress (4 dS m^− 1^) and high salinity stress (8 dS m^− 1^). T-RFs are shown on the left side and the identities of the T-RFs were based on in-silico prediction of 16S rRNA sequences of isolates and clones from the study

There is a general difference in the pattern of dominance of ribotypes or T-RFs between the salt-sensitive and salt-tolerant rice cultivars when they were grown under different salt stress conditions (Fig. 5, Additional file 7: Figure S4, Additional file 8: Figure S5). As observed in HaeIII, HhaI and partly in DdeI T-RFLP profiles, the salt-sensitive cultivar, IR29, have a more even abundance between a few ribotypes under normal condition. When salt stress is induced into the soil, there is a major shift of abundance to a single ribotype, both observed at 4 dS m^− 1^ and at 8 dS m^− 1^. For instance, in HhaI profile, there is almost an equal dominance of ribotype 327 (*Microbacterium*, *Flavobacterium*, *Pantoea*, and *Kosakonia*), 322 (*Enterobacter*) and partially by ribotype 353. At 4 and 8 dS m^− 1^, the bacterial community became dominated by a single ribotype, 322 and 327, respectively. The same could be described in HaeIII profile. On the other hand, the seed endophytes of the moderately salt-tolerant and the highly salt-tolerant cultivars, IC32 and IC37, responded differently. In general, the dominant ribotype(s) during non-stress condition was also the dominant ribotype under salt stress conditions. For IC32, it is dominated by ribotype 115 (*Flavobacterium*, *Pantoea*, *Microbacterium*, *Kosakonia*) in both 0 and 8 dS m^− 1^, and partly at 4 dS m^− 1^ as shown in HaeIII profile. In HhaI profile, IC32 is also dominated by ribotype 327 (*Microbacterium*, *Flavobacterium*, *Pantoea*, *Kosakonia*) and secondly by 322 (*Enterobacter*) which were also the dominant ribotypes under salt stress conditions. The same general pattern was also observed for the highly salt-tolerant cultivar, IC37.

Aside from general fluctuations in the relative abundance of the bacterial endophytes comprising the community structure of seeds due to salinity in the soil, the less dominant (abundant) members of the community may also become undetected under salt stress condition. There are also several less dominant ribotypes that appear during induced salt stress. These were observed for all the cultivars in different restriction enzyme profiles. For example, in HaeIII profile for IR29 (Fig. 1), ribotypes 140 and 145 were detected under normal conditions but disappeared under salt stress conditions. On the contrary, ribotype 55 was not detected under normal condition but was detected at 8 dS m^− 1^.

**Fig. 6:**
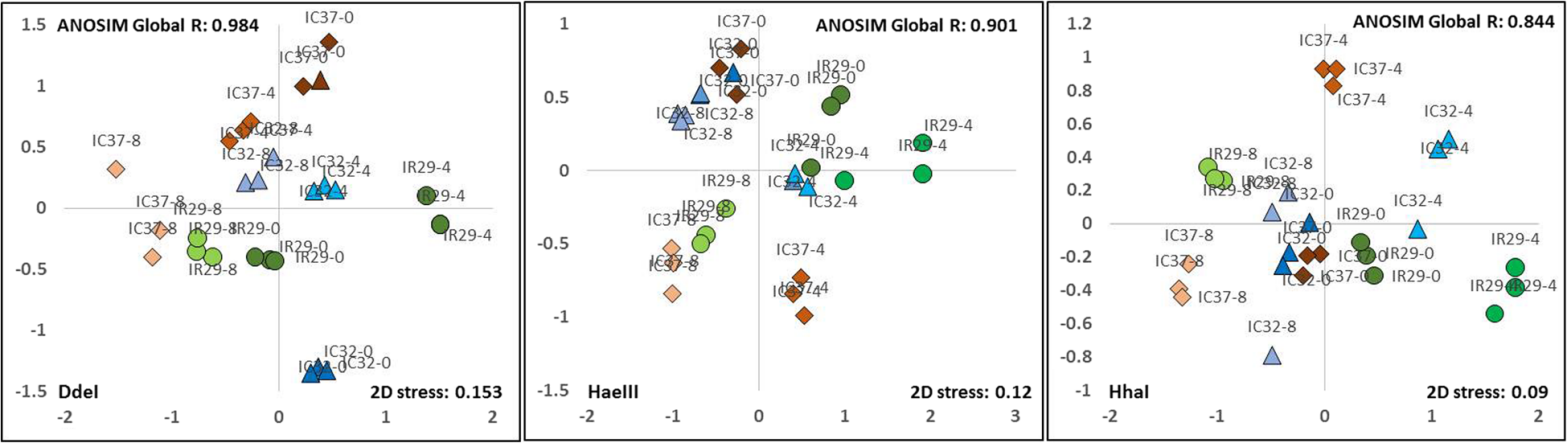
NMDS ordination of seed bacterial endophytes in different T-RFLP profiles from three rice cultivars with varying degree of tolerance: IR29 – salt sensitive, IC32 – moderately tolerant, IC37 – highly tolerant grown under normal condition (0 dS m^− 1^), moderate salinity stress (4 dS m^− 1^) and high salinity stress (8 dS m^− 1^). Global R values were calculated through Analysis of Similarity (ANOSIM). P-values of ANOSIM for DdeI, HaeIII and HhaI are significant at P(%) = 0.1

NMDS analysis of the bacterial communities in the three rice cultivars grown in different salt stress conditions showed the interaction of both genotype and soil salinity as major factors influencing the community structure of the seed endophytes (Fig. 6). Clustering of the seed bacterial community showed a general pattern as based on specific cultivar grown under certain salt stress conditions followed by salinity level of the soil. *P*-values of ANOSIM for DdeI, HaeIII and HhaI are significant at P(%) = 0.1 (Additional file 9).

## Discussion

This study is an attempt to understand the ecology, community structure and diversity of rice (*Oryza sativa* L. ssp. *indica*) seed endophytes. We used culture-dependent and culture-independent approaches to gain a complex picture of the endophytic bacterial diversity as affected by genotype, physiology and host phylogenetic relatedness of the rice host. Using T-RFLP, we found that the community structure and diversity of rice seed endophytes is characterized by its common and genotype-specific endophytes (Fig. 3, Additional file 3: Figure S1 and Additional file 4: Figure S2). These common endophytes represent a potential ‘core endophytic microbiota’ that is present in all the cultivars irrespective of the host plant’s inherent characteristics. Host factors contribute to the endophytic bacterial communities of rice seeds as genotype, physiological adaptation to salt stress and host phylogenetic relationships play a role in shaping endophytic communities. (Fig. 4 and Additional file 5: Figure S3). When grown under different salt stress conditions, the effect of soil salinity was observed to affect seed bacterial endophytes, as relative abundances of ribotypes fluctuate under different soil salinity condition (Fig. 5, Additional file 7: Figure S4 and Additional file 8: Figure S5) and bacterial community cluster based on genotype and salinity (Fig. 6).

### The major groups of seed bacterial endophytes across rice cultivars

The community structure and diversity of bacterial endophytes found in the different seeds seem to mainly depend on the specific cultivar or genotype of the host and is shaped by multiple factors ultimately resulting in relatively diverse endophytic community uniquely distinct to that of the host. Each rice cultivar has their own set of bacteria developing into a relatively rich community with even or a more dominant groups and varying diversity. The structure and diversity may be unique to each cultivar, but the major groups of seed bacterial endophytes seem to follow a general trend in terms of proportions. Gammaproteobacteria accounts for the majority of bacterial endophytes (63%) followed by Betaproteobacteria (15%), Actinobacteria (14%) and a few representatives of Alphaproteobacteria (4%) and Bacilli (4%). These major groups of bacteria were also observed in other studies of endophytes [15] and also in the rice roots [35]. Meta-analysis of sequenced bacteria also shows that bacterial endophytes are largely represented among four major bacterial phyla namely Proteobacteria, Actinobacteria, Firmicutes and Bacteroidetes [36].

### There are core bacterial endophytic microbiota existing among the rice indica subspecies

Rice cultivars in this study belong to *Oryza sativa ssp. indica*, all of which have undergone repeated inbreeding in order to create pure inbred lines from the original hybrids. They are products of human crossbreeding methods with the goal of attaining desirable traits, usually salt tolerance and high yield, of which the parents also belong to the indica subspecies (IRRI, RDA). Despite divergent selection and breeding with the resulting differences in genotypes, physiological tolerance to salt stress and branching parental lines, the indica seeds have maintained a shared set of associated bacteria that is potentially present in other indica cultivars and related rice species. T-RFLP analysis of seed endophytes shows that there are several genera of bacterial endophytes, each genus potentially contains a single bacterial species common to all rice cultivars. These include *Xanthomonas*, *Curtobacterium*, *Herbaspirillum*, *Microbacterium*, *Delftia* and *Stenotrophomonas*. This was also consistent in the cultured bacterial isolates particularly for *Flavobacterium*, *Xanthomonas* and *Microbacterium* where similar bacterial strains were isolated from many of the cultivars. Characterization and genetic fingerprinting of these culturable bacterial endophytes from the different rice cultivars point out in a few individual bacterial species with strains isolated from all the rice cultivars [37]. The presence of these shared or common bacterial species also indicate core microbial groups that are conserved and vertically transmitted throughout generations from their ancestral lines to the modern host cultivar [6]. These core microbial communities were also observed as common rice seed endophytes in other studies: *Xanthomonas* [38], *Curtobacterium* [8, 39], *Microbacterium*, *Stenotrophomonas* [8]; but may not be exclusively found in the seeds as they also colonize other endosphere niches. The occurrence of similar bacterial communities among *indica* subspecies has been partly observed in comparison with other subspecies of *Oryza sativa* [14], and that there is vertical transmission of bacterial endophytes from seeds to seedlings [8] and different lineages of host plants [6] as well as occurrence of strongly associated endophytes in rice leaf [40]. The results of this study where core microbial groups are present in all the indica cultivars may indicate a possibility for a high degree of transmission of endophytes from seed to mature host plant and from ancestral host plants to many modern indica lines. This accounts for the persistence and conservation of core endophytes in the rice rather than host selection from the soil. Transmission of endophytes from seed to seed of the next generation host plants were also partially observed in this study. Groups of bacterial endophytes detected in the original seeds of IR29, IC32 and IC37 were also the groups responding to changes in soil salinity when the seeds were recultivated, then salt stress was imposed. In general, the indica seeds contain the same groups of bacterial endophytes whose relative abundances shift in response to changing environmental conditions. Some of these groups are potentially core microbial endophytes of rice. The persistent presence and vertical transmission of endophytes also suggest an evolved form of mutualism or benign parasitism with their host [41]. The general abundance of core microbial groups in all cultivars also suggests their potential functional and coevolutionary importance across cultivars.

Endophytic diversity in the different endosphere regions has been reported in rice cultivars but to our own knowledge, this is the first report to suggest the existence of a core microbiota in *Oryza sativa ssp. indica* residing in the seeds. In this study, core endophytes are bacterial groups that are usually present in indica seeds regardless of genotype, host phylogenetic relatedness and physiological tolerance to salt stress conditions. These core endophytes may also show high association to rice and could be continually transmitted and conserved in the rice host. This indicates the need to study mechanisms of interaction of core endophytes and the rice host in order to understand how endophytes persist in the plant endosphere and continually modulate plant growth and health. Elucidating plant-endophyte interaction would also allow us to utilize endophytes as bio-inoculants to naturally and sustainably promote growth and yield of important agricultural crops such as rice.

### The bacterial community and diversity of rice seeds are influenced by its host’s genotype, physiological adaptation to salinity stress and host phylogenetic relatedness

The bacterial endophytic community and diversity are determined by multiple factors, both biotic and abiotic, ultimately shaping the community structure, population, diversity and inseparably, the functions of the plant’s microbiota [42]. An important factor that needs great consideration is the host plant: its genotype, physiology, developmental stages and endophyte-pathogen-host interaction among other factors yet to be considered. Despite the presumed importance of plant host factors, studies on the direct impact of these factors are limited and the overall knowledge of the interaction of host plant factors, abiotic factors and their influence on diversity and community structure of resident endophytes is far from complete. This study showed that under similar normal environmental conditions, the seed bacterial endophytes of rice are shaped mainly by the host genotype, physiological adaptation to salt stress and partly by host phylogenetic relatedness. Across different studies, genotype is a key factor determining composition of the different bacterial communities across cultivars of the same species of host [14], among phylogenetically related host [6], among differing conditions of the host plants [43] and across different plant species affected by temporal and spatial environmental variations [44]. The type of host plant, in itself, has a cumulative effect on the community structure and diversity of endophytes. This is observed even between highly related host plants within the indica subspecies where each cultivar develops their set of bacterial community related to other cultivars but uniquely distinct to one another. Furthermore, the effects of physiological differences on seed endophyte diversity and structure between related host plants have not been thoroughly investigated. Our study shows that physiological tolerance to salinity of the different cultivars has an additive impact on the community structure of rice seeds. Host physiological differences seem to also favor bacterial endophytes tuned to the host’s physiology as in the case of pathogen resistant and susceptible potato [45] and high nutrient use efficiency rice cultivar against lower nutrient use efficiency cultivars [14]. In this study, there are a few T-RFs potentially common to salt-tolerant cultivars only. However, the higher degree of occurrence of common ribotypes regardless of physiological differences suggests that most of the endophytes inhabiting the seed endosphere are adapted to high osmotic pressure [12] and are also salt-tolerant generally capable of surviving higher than or equal to 6% salt concentration [37].

### Bacterial endophytes residing in the seeds respond to the changing soil salinity conditions

Salt stress is not a singular stress but rather composed of interconnected factors affecting plants. It causes salt toxicity which is the disproportionate presence of Na^+^ in both cellular and extracellular compartments of plants [17], osmotic problems [46], induction and accumulation of reactive oxygen species [47], and interference in terms of essential nutrient uptake [46]. These interconnected conditions under salt stress could directly or indirectly affect the endophytes of rice and consequently also determine the seed bacterial community. In addition, under salt stress conditions, seed bacterial endophytes residing in the endosphere of rice are shaped by both the genotype of the host plant and soil salinity level. The seed bacterial endophytes respond to their changing environmental condition brought about by soil salinity and its effects on the physiology of the host plants. This was also observed in plants growing in specific soil and environmental condition causing endophytes to respond and become competent [8] as well as in *Phragmites* and their archaeal endophytic communities [48]. The endophytic bacterial communities of roots have also dramatically changed in terms of diversity indices, community structure and abundance as salt stress impairs the growth of *Medicago truncatula* [49]. These studies show that endophytes are influenced by their host physiology as the hosts respond to different environmental conditions, especially with regards to the soil. Different soils can directly influence the host plants and can also be a source of endophytes for the plants. As host plants are cultivated in different soils, these may lead to changes in host endophytic composition but seed-borne endophytes respond to these changes [8] that potentially allow them to be transmitted and conserved through the seeds [6]. Seed endophytes are also partially buffered by differences in soil used [50] allowing transmission of seed-borne endophytes even in different soil or ecogeographic locations.

Some endophytic bacterial communities also become more prominent and dominant under salt stress conditions. This indicates their potential importance or adaptive competence over other bacterial groups as they become key players, directly influencing survival of their host plant under stress conditions. A few studies on endophytes have also shown that there are community changes associated with specific stress conditions [8, 44, 45]. In this study, there is a shift in dominance in terms of abundance to certain groups including *Flavobacterium*, *Pantoea*, *Curtobacterium*, *Microbacterium*, *Kosakonia* and *Enterobacter*. There is also a consistent presence of *Stenotrophomonas* and *Xanthomonas* though their abundance is somewhat maintained in different levels of salinity. Characterization of the isolates [37] showed that *Flavobacterium*, *Pantoea*, *Kosakonia* and *Microbacterium* strains are distinguished by their salt tolerance, osmotic tolerance and IAA production when correlated with other endophytic groups and their physiological characteristics. Yaish et al. [49] showed that some endophytes appear under salt stress conditions including members of *Flavobacteria*, *Streptomycetales*, *Enterobacter* and *Pseudomonas* in the roots of *Medicago truncatula*. In our study, the dominant bacterial groups under salt stress were already present under normal conditions albeit their abundance was of lower magnitude. The effect of the environmental stimulus is also observed in the dominance of fungal endophytic community under habitat induced stress [51].

## Conclusions

The structure of endophytic bacterial communities in the seed endosphere of indica rice is shaped by their hosts’ genotype, physiological adaptation to salt stress and partly by host phylogenetic relationships. Community composition of all the rice cultivars in this study also suggests the presence of potential core microbial groups associated with rice and they are most probably transmitted through the seeds. Under soil salinity conditions, bacterial diversity and dominance fluctuate in the salt-sensitive and salt-tolerant cultivars where endophytic groups belonging to *Flavobacterium*, *Pantoea*, *Curtobacterium*, *Microbacterium*, *Kosakonia* and *Enterobacter* generally become the dominant groups while *Stenotrophomonas* and *Xanthomonas* seem to maintain their abundance under normal and salt-stress conditions. This study shows that the endophytic communities of the rice seeds are conditioned by both the host plant and by their external environment.

## Additional files

Additional file 1: **Table S1.** Characteristics of the six (6) rice cultivars (*Oryza sativa* L. ssp. *indica*) used to assess bacterial community associated with the seeds. (DOCX 15 kb)

Additional file 2: **Table S2.** Bacterial population profiles in the seeds of salt-tolerant and salt-sensitive cultivars of *Oryza sativa ssp. indica*. (DOCX 16 kb)

Additional file 3: **Figure S1.** Heat map, relative abundance and total abundance of ribotypes (T-RF’s) present in the different seeds rice cultivars after digestion with DdeI. IR29 is a salt-sensitive cultivar, FL478, IC27, IC31 and IC32 are moderately salt-tolerant cultivars and IC37 is highly salt-tolerant rice cultivar. T-RFs are on the left side of the heat map and the identities of the T-RFs were based on in-silico prediction of 16S rRNA sequences of isolates and clones from the study. The heat map is arranged according to decreasing overall abundance. (JPEG 137 kb)

Additional file 4: **Figure S2.** Heat map, relative abundance and total abundance of ribotypes (T-RF’s) present in the different seeds rice cultivars after digestion with Hha I. IR29 is a salt-sensitive cultivar, FL478, IC27, IC31 and IC32 are moderately salt-tolerant cultivars and IC37 is highly salt-tolerant rice cultivar. T-RFs are on the left side of the heat map and the identities of the T-RFs were based on in-silico prediction of 16S rRNA sequences of isolates and clones from the study. The heat map is arranged according to decreasing overall abundance. (JPEG 135 kb)

Additional file 5: **Figure S3.** Cluster analysis of cultivars based on Bray-Curtis similarities of the endophytic bacterial community T-RFLP data after digestion with restriction enzymes Dde I, Hae III and Hha I. (JPEG 70 kb)

Additional file 6: **Table S3.** Analysis of similarities (ANOSIM) of the seed endophytic bacterial community of the different indica rice cultivars showing global and pairwise test. (DOCX 14 kb)

Additional file 7: **Figure S4.** Heat map, relative abundance and total abundance of ribotypes (T-RF’s) after DdeI digestion present in the seeds of three rice cultivars based on their tolerance to salt: IR29 – salt sensitive, IC32 – moderately tolerant, IC37 – highly tolerant, grown under normal condition (0 dS m^− 1^), moderate salinity stress (4 dS m^− 1^) and high salinity stress (8 dS m^− 1^). (JPEG 129 kb)

Additional file 8: **Figure S5.** Heat map, relative abundance and total abundance of ribotypes (T-RF’s) after HhaI digestion present in the seeds of three rice cultivars based on their tolerance to salt: IR29 – salt sensitive, IC32 – moderately tolerant, IC37 – highly tolerant, grown under normal condition (0 dS m^− 1^), moderate salinity stress (4 dS m^− 1^) and high salinity stress (8 dS m^− 1^). (JPEG 110 kb)

Additional file 9: **Table S4.** Analysis of similarities (ANOSIM) of the seed endophytic bacterial community in salt-sensitive (IR29), moderately salt-tolerant (IC32) and highly salt-tolerant (IC37) rice cultivars grown under normal condition (0 dS/m), moderate salinity (4 dS/m) and high soil salinity (8 dS/m). (DOCX 14 kb)
