# Supplementary material for: The influence of host genotype and salt stress on the seed endophytic community of salt-sensitive and salt-tolerant rice cultivars
Source: BMC Plant Biol. 2018 Mar 27;18:51. doi: 10.1186/s12870-018-1261-1 (PMC5870378; doi:10.1186/s12870-018-1261-1)
Supplement: Supplementary file 1 — Table S1. Characteristics of the six (6) rice cultivars (Oryza sativa L. ssp. indica) used to assess bacterial community associated with the seeds. (DOCX 15 kb) [file 12870_2018_1261_MOESM1_ESM.docx]

**Table S1** Characteristics of the six (6) rice cultivars (*Oryza sativa* L. ssp. *indica*) used to assess bacterial community associated with the seeds

| Rice Cultivar | Parental Lines | Harvest Date | Stem Height | Salinity Tolerance | |
| --- | --- | --- | --- | --- | --- |
|  |  |  | cm | Early growth | Mature plant |
| IR29 | IR833-6-2-1-1 I 11 (1561-149-1) / /1R1737 | 21 August 2014 | 78 | Weak | Weak |
| FL478 | IR29/POKKALI B | 18 August 2014 | 61 | Strong | Moderate |
| IC27 | IR42/IR 4630-22-2-5-1-3 | 05 September 2014 | 82 | Moderate | Moderate |
| IC31 | IR 4630-22-2-5-1-3/NONA BOKRA | 15 August 2014 | 71 | Strong | Moderate |
| IC32 | AT 401/IR31868-64-2-3-3-3 | 18 August 2014 | 69 | Strong | Moderate |
| IC37 | IR 73012-137-2-2-2/PSB RC 10 (IR 50404-57-2-2-3 | 22 August 2014 | 67 | Strong | Strong |

Source: International Rice Research Institute, Philippines (IRRI); Rural Development Administration, Korea (RDA).
