# Supplementary material for: The influence of host genotype and salt stress on the seed endophytic community of salt-sensitive and salt-tolerant rice cultivars
Source: BMC Plant Biol. 2018 Mar 27;18:51. doi: 10.1186/s12870-018-1261-1 (PMC5870378; doi:10.1186/s12870-018-1261-1)
Supplement: Supplementary file 2 — Table S2. Bacterial population profiles in the seeds of salt-tolerant and salt-sensitive cultivars of Oryza sativa ssp. indica. (DOCX 16 kb) [file 12870_2018_1261_MOESM2_ESM.docx]

**Table S2** Bacterial population profiles in the seeds of salt-tolerant and salt-sensitive cultivars of *Oryza sativa* ssp*. indica.*

| Rice cultivar | Population | Gram (-) | Gram (+) | Cultivable isolates |
| --- | --- | --- | --- | --- |
|  | CFU g^-1^ | % | % |  |
| IR29 (SS) | 5.46±0.09 ^c^ | 42.9 | 57.1 | 7 |
| FL478 (MT) | 5.31±0.02 ^c^ | 57.1 | 42.9 | 7 |
| IC27 (MT) | 6.31±0.04 ^b^ | 100.0 | 0.0 | 4 |
| IC31 (MT) | 5.64±0.04 ^c^ | 90.0 | 10.0 | 10 |
| IC32 (MT) | 6.52±0.04 ^a^ | 71.4 | 28.5 | 14 |
| IC37 (ST) | 4.24±0.04 ^c^ | 0.00 | 100.0 | 7 |

Population is presented as means ± SE (standard error) from three replicates. Means with the same letter are not statistically significant. (SS) salt-sensitive cultivar, (MT) moderately tolerant cultivar, (ST) strongly tolerant cultivar.
