# Supplementary figures and images for: The influence of host genotype and salt stress on the seed endophytic community of salt-sensitive and salt-tolerant rice cultivars
Source: BMC Plant Biol. 2018 Mar 27;18:51. doi: 10.1186/s12870-018-1261-1 (PMC5870378; doi:10.1186/s12870-018-1261-1)

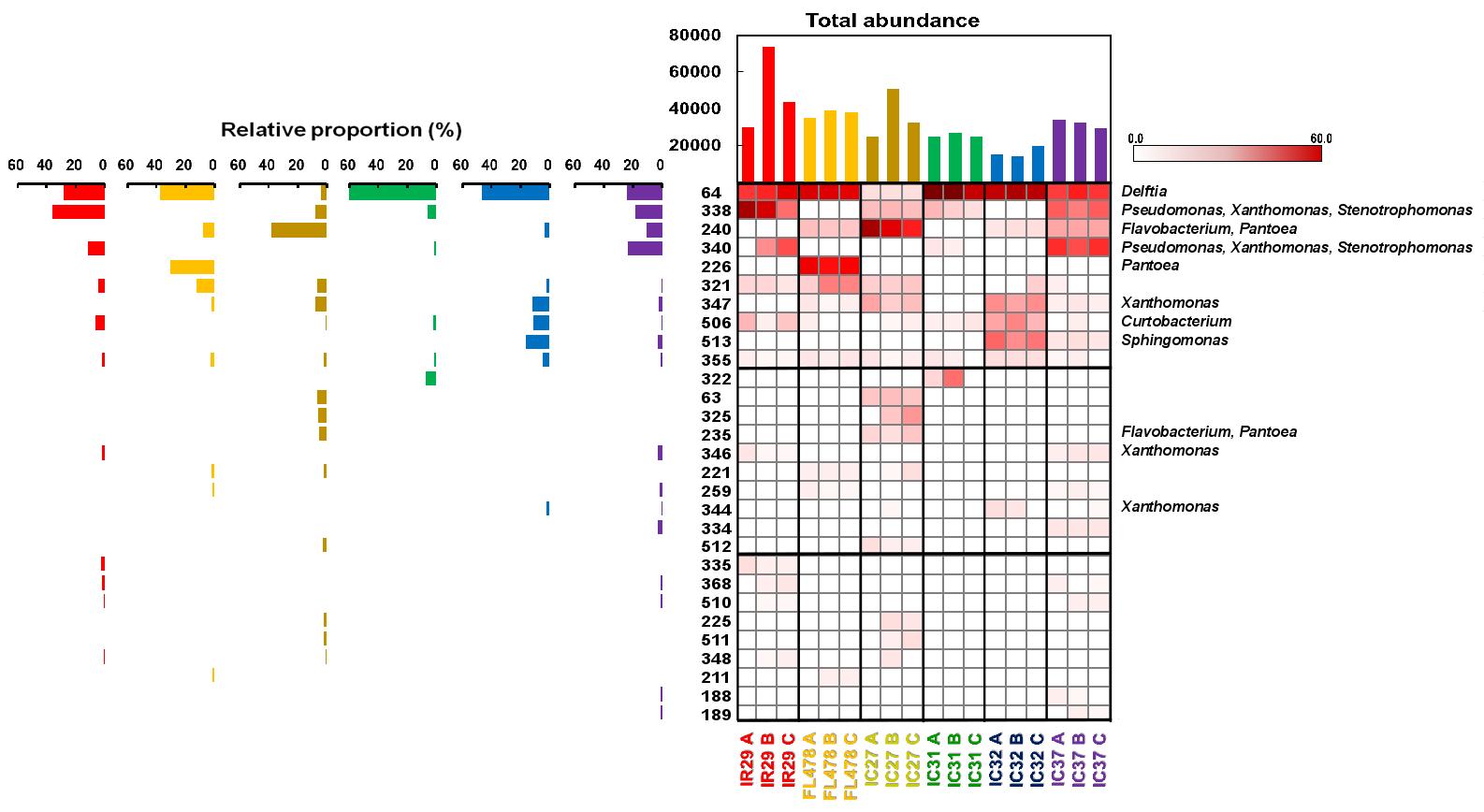

Supplement: Supplementary file 3 — Figure S1. Heat map, relative abundance and total abundance of ribotypes (T-RF’s) present in the different seeds rice cultivars after digestion with DdeI. IR29 is a salt-sensitive cultivar, FL478, IC27, IC31 and IC32 are moderately salt-tolerant cultivars and IC37 is highly salt-tolerant rice cultivar. T-RFs are on the left side of the heat map and the identities of the T-RFs were based on in-silico prediction of 16S rRNA sequences of isolates and clones from the study. The heat map is arranged according to decreasing overall abundance. (JPEG 137 kb) [file 12870_2018_1261_MOESM3_ESM.jpg]

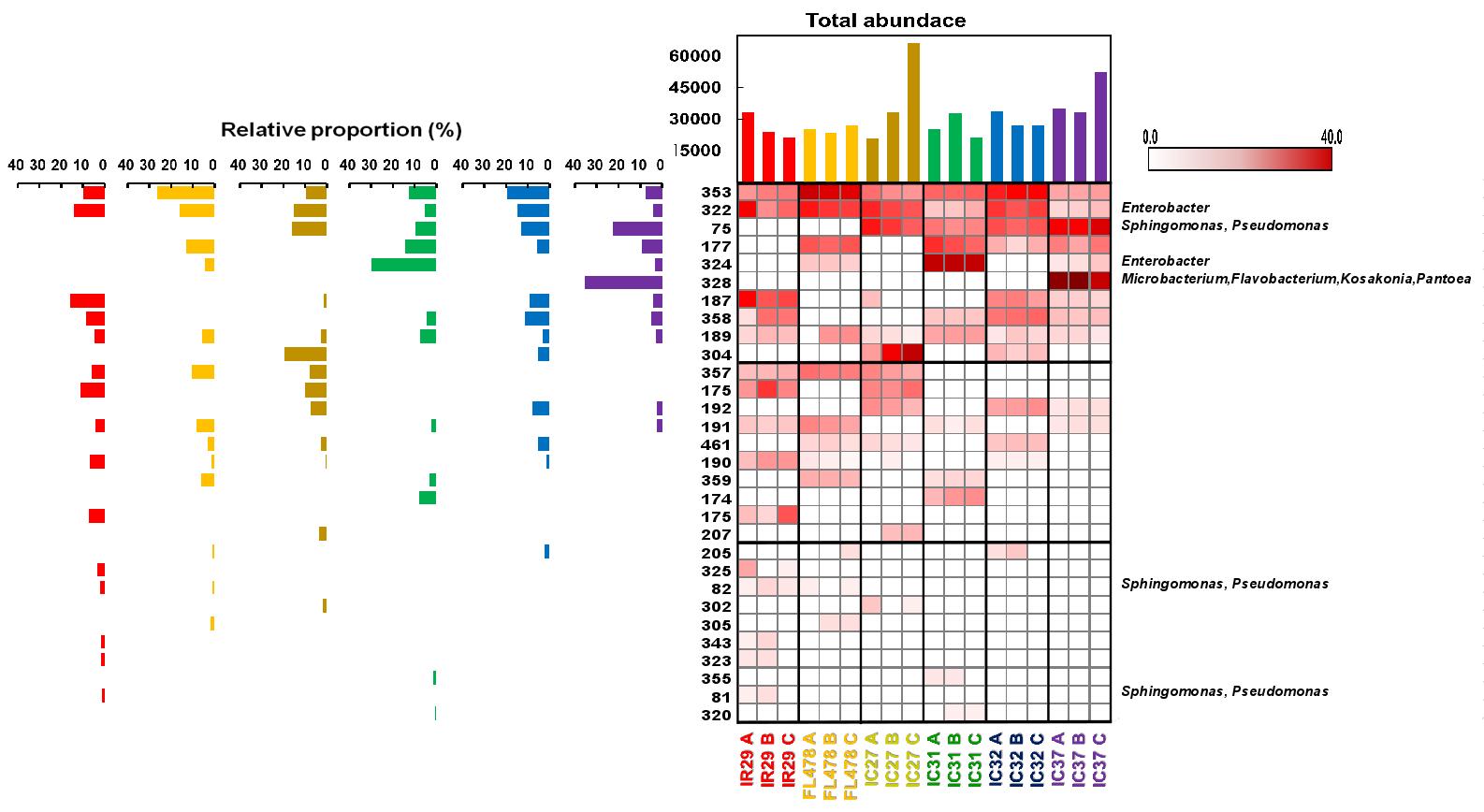

Supplement: Supplementary file 4 — Figure S2. Heat map, relative abundance and total abundance of ribotypes (T-RF’s) present in the different seeds rice cultivars after digestion with Hha I. IR29 is a salt-sensitive cultivar, FL478, IC27, IC31 and IC32 are moderately salt-tolerant cultivars and IC37 is highly salt-tolerant rice cultivar. T-RFs are on the left side of the heat map and the identities of the T-RFs were based on in-silico prediction of 16S rRNA sequences of isolates and clones from the study. The heat map is arranged according to decreasing overall abundance. (JPEG 135 kb) [file 12870_2018_1261_MOESM4_ESM.jpg]

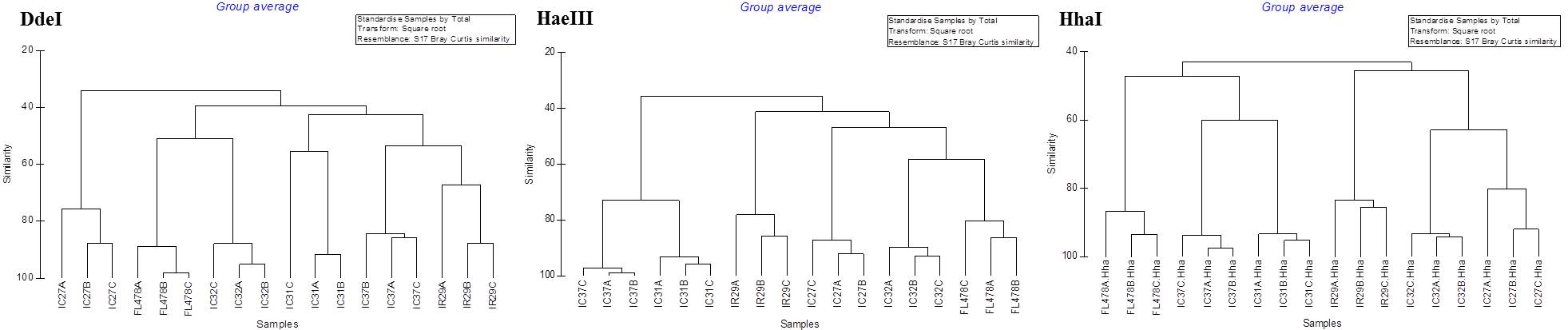

Supplement: Supplementary file 5 — Figure S3. Cluster analysis of cultivars based on Bray-Curtis similarities of the endophytic bacterial community T-RFLP data after digestion with restriction enzymes Dde I, Hae III and Hha I. (JPEG 70 kb) [file 12870_2018_1261_MOESM5_ESM.jpg]

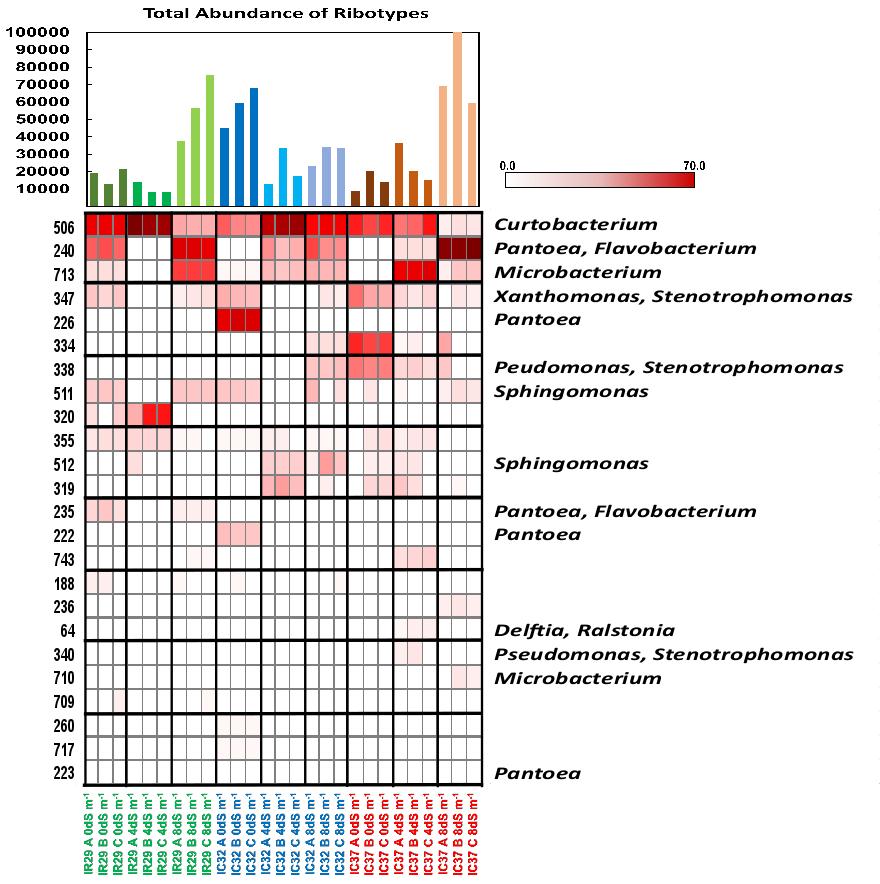

Supplement: Supplementary file 7 — Figure S4. Heat map, relative abundance and total abundance of ribotypes (T-RF’s) after DdeI digestion present in the seeds of three rice cultivars based on their tolerance to salt: IR29 – salt sensitive, IC32 – moderately tolerant, IC37 – highly tolerant, grown under normal condition (0 dS m− 1), moderate salinity stress (4 dS m− 1) and high salinity stress (8 dS m− 1). (JPEG 129 kb) [file 12870_2018_1261_MOESM7_ESM.jpg]

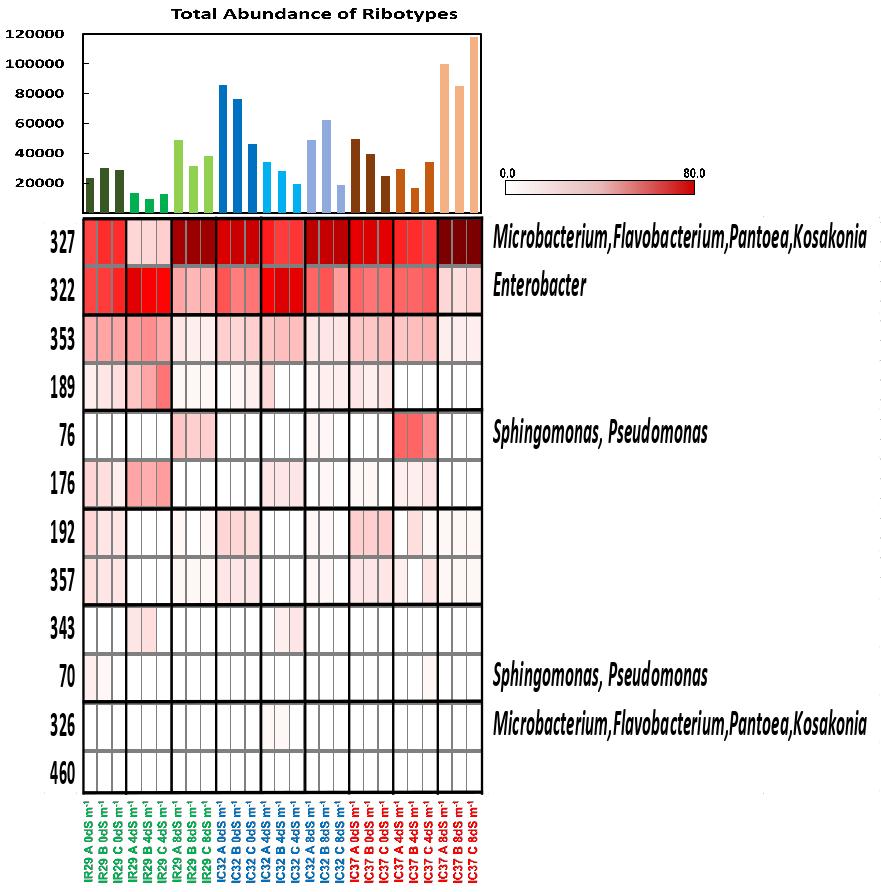

Supplement: Supplementary file 8 — Figure S5. Heat map, relative abundance and total abundance of ribotypes (T-RF’s) after HhaI digestion present in the seeds of three rice cultivars based on their tolerance to salt: IR29 – salt sensitive, IC32 – moderately tolerant, IC37 – highly tolerant, grown under normal condition (0 dS m− 1), moderate salinity stress (4 dS m− 1) and high salinity stress (8 dS m− 1). (JPEG 110 kb) [file 12870_2018_1261_MOESM8_ESM.jpg]
