# Supplementary material for: The influence of host genotype and salt stress on the seed endophytic community of salt-sensitive and salt-tolerant rice cultivars
Source: BMC Plant Biol. 2018 Mar 27;18:51. doi: 10.1186/s12870-018-1261-1 (PMC5870378; doi:10.1186/s12870-018-1261-1)
Supplement: Supplementary file 6 — Table S3. Analysis of similarities (ANOSIM) of the seed endophytic bacterial community of the different indica rice cultivars showing global and pairwise test. (DOCX 14 kb) [file 12870_2018_1261_MOESM6_ESM.docx]

Table S3 Analysis of similarities (ANOSIM) of the seed endophytic bacterial community of the different indica rice cultivars showing global and pairwise test.

| Pairwise R Statistic | Restriction enzyme | | |
| --- | --- | --- | --- |
| Pairwise tests | DdeI | HaeIII | HhaI |
| IR29, FL478 | 1 | 1 | 1 |
| IR29, IC27 | 1 | 1 | 1 |
| IR29, IC31 | 1 | 1 | 1 |
| IR29, IC32 | 1 | 1 | 1 |
| IR29, IC37 | 1 | 1 | 1 |
| FL478, IC27 | 1 | 1 | 1 |
| FL478 ,IC31 | 1 | 1 | 1 |
| FL478 ,IC32 | 1 | 1 | 1 |
| FL478, IC37 | 1 | 1 | 1 |
| IC27, IC31 | 1 | 1 | 1 |
| IC27, IC32 | 1 | 1 | 1 |
| IC27, IC37 | 1 | 1 | 1 |
| IC31 ,IC32 | 1 | 1 | 1 |
| IC31, IC37 | 1 | 1 | 1 |
| IC32, IC37 | 1 | 1 | 1 |
| Global R | 0.989 | 1 | 1 |
| P (%) | 0.1 | 0.1 | 0.1 |
