# Supplementary material for: The influence of host genotype and salt stress on the seed endophytic community of salt-sensitive and salt-tolerant rice cultivars
Source: BMC Plant Biol. 2018 Mar 27;18:51. doi: 10.1186/s12870-018-1261-1 (PMC5870378; doi:10.1186/s12870-018-1261-1)
Supplement: Supplementary file 9 — Table S4. Analysis of similarities (ANOSIM) of the seed endophytic bacterial community in salt-sensitive (IR29), moderately salt-tolerant (IC32) and highly salt-tolerant (IC37) rice cultivars grown under normal condition (0 dS/m), moderate salinity (4 dS/m) and high soil salinity (8 dS/m). (DOCX 14 kb) [file 12870_2018_1261_MOESM9_ESM.docx]

Table S4 Analysis of similarities (ANOSIM) of the seed endophytic bacterial community in salt-sensitive (IR29), moderately salt-tolerant (IC32) and highly salt-tolerant (IC37) rice cultivars grown under normal condition (0 dS/m), moderate salinity (4 dS/m) and high soil salinity (8 dS/m).

| Pairwise R Statistic | Restriction enzyme | | |
| --- | --- | --- | --- |
| Pairwise tests | DdeI | HaeIII | HhaI |
| IR29, IC32 | 1 | 0.926 | 0.815 |
| IR29, IC37 | 0.926 | 0.988 | 1 |
| IC32, IC37 | 1 | 1 | 0.765 |
| Global R | 0.984 | 0.901 | 0.844 |
| P (%) | 0.1 | 0.1 | 0.1 |
|  |  |  |  |
| 0 dS/m, 4 dS/m | 1 | 0.914 | 1 |
| 0 dS/m, 8 dS/m | 1 | 1 | 0.827 |
| 4 dS/m, 8 dS/m | 1 | 1 | 1 |
| Global R | 1 | 0.981 | 0.931 |
| P (%) | 0.1 | 0.1 | 0.1 |
